# Towards an understanding of global brain data governance: ethical positions that underpin global brain data governance discourse

**DOI:** 10.3389/fdata.2023.1240660

**Published:** 2023-11-09

**Authors:** Paschal Ochang, Damian Eke, Bernd Carsten Stahl

**Affiliations:** ^1^Centre for Computing and Social Responsibility, De Montfort University, Leicester, United Kingdom; ^2^School of Computer Science, University of Nottingham, Nottingham, United Kingdom

**Keywords:** brain data, data governance, neuroethics, ethics, neurodata, ethical theories, ethical positions

## Abstract

**Introduction:**

The study of the brain continues to generate substantial volumes of data, commonly referred to as “big brain data,” which serves various purposes such as the treatment of brain-related diseases, the development of neurotechnological devices, and the training of algorithms. This big brain data, generated in different jurisdictions, is subject to distinct ethical and legal principles, giving rise to various ethical and legal concerns during collaborative efforts. Understanding these ethical and legal principles and concerns is crucial, as it catalyzes the development of a global governance framework, currently lacking in this field. While prior research has advocated for a contextual examination of brain data governance, such studies have been limited. Additionally, numerous challenges, issues, and concerns surround the development of a contextually informed brain data governance framework. Therefore, this study aims to bridge these gaps by exploring the ethical foundations that underlie contextual stakeholder discussions on brain data governance.

**Method:**

In this study we conducted a secondary analysis of interviews with 21 neuroscientists drafted from the International Brain Initiative (IBI), LATBrain Initiative and the Society of Neuroscientists of Africa (SONA) who are involved in various brain projects globally and employing ethical theories. Ethical theories provide the philosophical frameworks and principles that inform the development and implementation of data governance policies and practices.

**Results:**

The results of the study revealed various contextual ethical positions that underscore the ethical perspectives of neuroscientists engaged in brain data research globally.

**Discussion:**

This research highlights the multitude of challenges and deliberations inherent in the pursuit of a globally informed framework for governing brain data. Furthermore, it sheds light on several critical considerations that require thorough examination in advancing global brain data governance.

## 1. Introduction

Advances in neuroscience and the study of the brain have continued to generate large scale high-quality big brain datasets. These datasets which are essential for advancing neurotechnologies and developing new treatments are generated in different jurisdictions and consists of datasets from multiple disciplines, organisms, while also existing in multiple formats (Landhuis, 2017; Rommelfanger et al., 2018; Adams et al., 2020; Eke D. O. et al., 2021). This complexity and the sensitivity of brain data raises several challenges in the collection, processing and sharing of brain data. Some of these challenges include privacy, informed consent, security, confidentiality, and ownership. While the existence of an appropriate global governance mechanism for brain data would help to curtail some of these challenges, this has not been the case as currently a global framework for the governance of brain data is non-existent. This has given rise to calls for the development of an international data governance framework for brain data to foster data sharing and collaboration. The development of such of a governance framework should be culturally informed (Ienca et al., 2022) while acknowledging the pluralistic nature of ethical and legal principles that exist in various jurisdictions (Eke D. O. et al., 2021; Ienca et al., 2022). These ethical considerations and implications can provide tools for navigating the ethical and moral hurdles that exist in the management of brain data. Furthermore, acknowledgment of ethical principles and the embedding of ethics in policies and practices can promote large scale collaboration and data sharing in neuroscience projects (Stahl et al., 2018).

While the development of a global framework that is culturally informed will advance collaborations, several challenges currently exist in the implementation of such global frameworks. These challenges span across technical, regulatory, and ethical boundaries which influence the collection, processing, sharing and storage of brain data. Although there is a current acknowledgment of the importance of ethical considerations in the governance of brain data, various ethical and legal principles which influence brain data governance currently exist as identified in our previous study (Ochang et al., 2022). These principles such as privacy and consent are very visible in discussions (but multidimensional) while other principles and concepts such as neurorights and data retention and destruction are still less visible which shows that more work has to be done on their global conceptualisation and visibility. Also, discussions around the applicability of the principles that exist in the governance landscape are very multidimensional which calls for standardisation, agreements, and clear guidelines. These are but a few observations when exploring the ethical and legal landscape of brain data governance which can create hurdles in developing a governance framework.

Furthermore, as the boundaries between jurisdictions and legal systems (e.g., GDPR and HIPAA) are mostly territorial while moral and ethical perceptions are proving to be inconsistent (Friedman, 1969; Stahl, 2012) varying between societies, pluralism in ethics and legal principles will continue to influence brain research (Emerging Issues Task Force and International Neuroethics Society, 2019; Eke D. O. et al., 2021). Researchers hailing from different geographical regions could face challenges in comprehending the prerequisites for legal and ethical reciprocity when sharing brain data. Moreover, those accessing data from diverse origins, possibly spanning various jurisdictions, might lack clarity regarding whether de-identified, coded, unlinked, or pseudonymised data holds the same equivalence as reversibly anonymised data (Dove, 2015). Furthermore, although universal declarations such as the Universal Declaration of Human Rights (UDHR) (United Nations General Assembly, 1949) attempt to generalise ethical and legal principles which should be upheld, no declaration will ever be exhaustive in guiding the practices of key actors in brain data research. This is because universally accepted declarations which embody ethical and legal principles will always go hand in hand with the cultural and moral diversity of various regions. Also storing big brain datasets across multiple repositories and infrastructure raises technical challenges due to the fact that brain data researchers are no longer dealing with terabytes but petabytes of data due to the large-scale datasets generated by a few minutes of neural activity (Landhuis, 2017; Ochang et al., 2022). These are some of the challenges that exist in relation to developing a global governance framework.

Although recommendations have been made regarding developing a responsible framework (Fothergill et al., 2019) that is culturally informed, practical steps to develop such a framework using the perceptions of key stakeholders conducting brain research in different regions to understand their contextual perceptions has been limited. Also, neuroethical approaches, as illustrated by Farah (2015), often lack direct integration with data governance. Similarly, data governance research, as noted by Nielsen (2017), tends to overlook ethical dimensions. In developing a global framework for the governance of brain data that is culturally informed, various contextual challenges, issues, and concerns (regulatory, ethical, practical, and technical) must be understood. Such governance frameworks should also be dynamically responsive to the peculiar issues and challenges in the conduct of brain data research in various regions. In the current landscape of brain data governance which embodies several ethical and legal principles, these principles are underpinned by various ethical positions when applied in practice. When applied in practice they generate different issues and concerns which might be peculiar or contextual to the region of application. They also generate various standpoints, recommendations, and justifications. However, a clear understanding of these ethical positions of key stakeholders which can provide theoretical and practical insights for advancing a contextually aware global brain data governance framework, especially when applying current governance principles is currently lacking. This is one of the motivations for undertaking this study.

It is here that we situate our justification and research question which asks *what ethical positions underpin current brain data governance discourse?* In the application of ethical and legal principles, stakeholders are bound to make moral justifications based on, for example, virtues, duties, consequences, or what they consider the greater good. This generates various ethical insights, perspectives and recommendations around the issues and concerns inherent in current principles by stakeholders in different regions which can only be understood by attempting to explore ethical positions. Other factors that have influenced this study include the need to capture the insights of brain data researchers in regions, including Africa, thereby promoting inclusivity in current discussions. Having spoken to key neuroscientists to understand the application of ethical and legal principles in our previous research, this paper attempts to find out what ethical positions can be found in their practices especially around duties, virtues, consequences, and the need to agree on guidelines, regulations, and other binding practices.

To the best of our knowledge this is the first study which attempts to understand global brain data governance by bringing together neuroscientists with a global representation to provide discussions which are underpinned by various ethical positions to provide insights, justifications, and recommendations. The findings of the study revealled various issues, concerns and recommendations which are supported by various deontological, consequentialist and virtue positions. Some of the insights provide reasons to endorse and comply with fundamental laws, institutions and principles which are underpinned by the social contract theory.

This paper makes a dual contribution to the existing body of knowledge. Firstly, it provides a unique perspective on brain data governance by elucidating theoretical frameworks within the context of brain data. This elucidation aids in comprehending the ethical stances of stakeholders in the realm of brain data governance. Secondly, the paper addresses the diversity within the brain data governance landscape by convening prominent researchers. It acts as a catalyst for discussions that reflect the diverse aspects of brain data governance.

## 2. Conceptual background

### 2.1. Brain data

Brain data is multidisciplinary, uniting researchers from diverse fields of study. The collaboration among these disciplines yields diverse brain data types through various techniques and modalities. This contributes to its inherent complexity, further compounded by data from multiple species and organisms (Landhuis, 2017; Abbott, 2020), culminating in extensive brain data often termed big brain data. Notably, a mere 20-minute recording of neural activity generates ~500 petabytes of data (Landhuis, 2017). This large volume underscores that scientists grapple not only with the intricacy of brain data but also its magnitude. These attributes also satisfy key characteristics of big data (volume, velocity, variety, and veracity) (L'Heureux et al., 2017; Fothergill et al., 2019) resulting to brain data being sometimes referred to as big brain data (Landhuis, 2017; Kellmeyer, 2018). Given its diverse nature, reflecting processes in both human and multiple specimen brains, the term “brain data” lacks precise conceptual clarity and is often used ambiguously. To enhance conceptual precision, this paper encompasses both human and animal brain data. Furthermore, the paper argues that brain data has transcended raw measurements to include derived data and metadata (data of data). Therefore, to provide conceptual clarity this paper refers to brain data as *data that directly or indirectly (including metadata) pertains to the brain structure, activity, and function of humans, animals, and other organisms*. Examples include Magnetic Resonance Imaging (MRI), Positron Emission Tomography (PET), Magnetoencephalography (MEG) and Near-Infrared Spectroscopy (NIRS) (Liu et al., 2006; Crosson et al., 2010) and metadata in brain datasets. The inclusion of metadata in the definition of brain data is important as metadata offers descriptive or structural information that provides context and details about brain data.

### 2.2. Brain data governance and its potential dimensions

**Figure 1:**
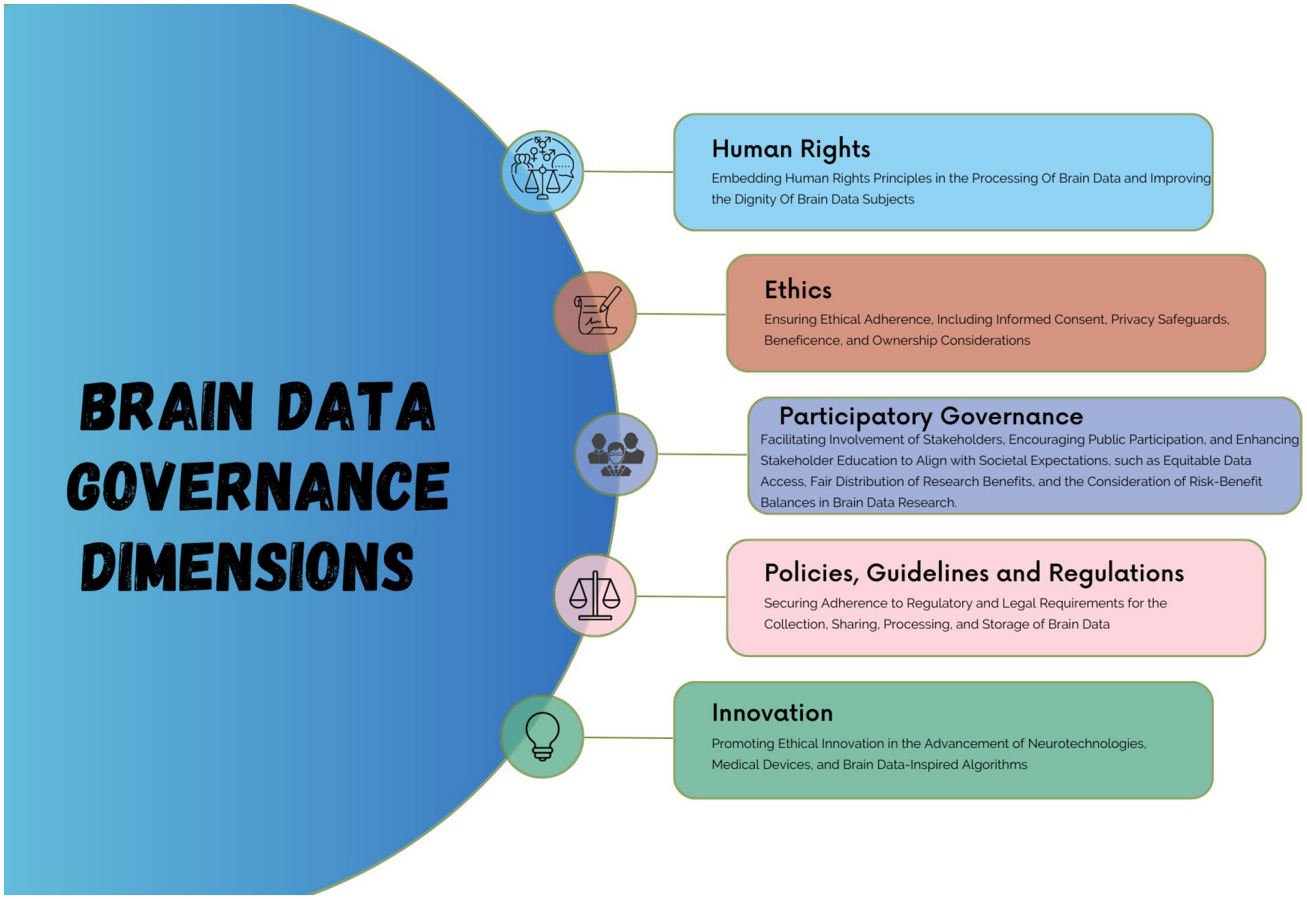
Brain data governance dimensions.

Brain data governance focuses on the policies, procedures, and systems that are established to oversee the collection, storage, use, sharing and management of brain data. It can be defined *as the policies and strategies that define responsibilities of accountable stewardship which include acquiring, aggregating, deidentifying, processing, curation, retention, deletion, use and the overall availability, usability, integrity, security, and privacy of data in alignment with ethical, legal, and social obligations* (Ochang et al., 2022). This definition acknowledges the ethical, legal, and social implications (ELSI) of collecting, processing, storing, and sharing brain data resulting in the need to consider key dimensions in the governance data that are different from other traditional forms of data. Key areas of brain data governance include ethics, policies, regulations and guidelines (binding laws), human rights, innovation (development of medical devices and neurotechnologies), and participatory governance (Ienca et al., 2022; Ochang et al., 2023) as shown in Figure 1. This also involves assessing technical provisions (e.g., security and data protection) to align with regulatory and ethical prerequisites, and prioritising alignment with research environments rather than corporate or conventional information systems environments.

### 2.3. The relationship between ethical theories and data governance

#### 2.3.1. Ethics

Ethics which stems from the Greek word “ethos” focuses on the moral foundations and reflections on which actions, behavioral judgements, and moral evaluations are made (Stahl, 2012; Fieser, 2018). Discussions around ethics span over millennia as ethics is firmly rooted in moral systems such as customs, religion, and law. The use of ethical theories to underpin current brain data governance discourse has been limited if not non-existent. While in the context of big data and information systems there is a reasonable application of ethical theories in an attempt to understand or advance data ethics (Mittelstadt and Floridi, 2016; Herschel and Miori, 2017; Hand, 2018; Bezuidenhout and Ratti, 2021). Ethical theories can provide explanatory mechanisms in the quest for understanding the ethical reasoning around the collection, use and sharing of data. For example, in the field of big data some researchers have used traditional ethical theories to analyse new power distributions in big data to show that the characteristics of big data shifts the nature of the ethics debate because it redefines power dynamics and the extent to which the element of free will exists in data utility (Zwitter, 2014). Also, traditional ethical theories used in data ethics provide an underpinning to the notion of moral responsibility which exists in the everchanging nature of our data ecosystem and has provided the foundation for advances in ethics such as in network ethics (Floridi, 2009), big data ethics (Herschel and Miori, 2017; Someh et al., 2019), computer and informatic ethics. Therefore, traditional ethical theories provide instruments to help in the framing of moral issues and recommendations that exist in the sharing and usage of data.

#### 2.3.2. Key ethical theories in data ethics

Ethical theories used in data ethics share a common property which is that they all provide instruments to make logical, reasoned, and persuasive arguments based on the principles of the theory in use (Herschel and Miori, 2017). Prominent ethical theories that are usually applied in data ethics often include Deontology (Kantianism or Kantian ethics), Consequentialism (utilitarianism), Virtue Ethics and Social Contract Theory (Stahl, 2012; Zwitter, 2014; Herschel and Miori, 2017). Deontology derived from the Greek word *deon* which stands for *duty* or *obligation* (Stahl, 2012; Farah, 2015) deontology focuses on the duty and obligations of the moral agent and duty that individuals have toward one another. Deontology focuses on the characteristics embedded in the action of the moral agent and attempts to evaluate the ethical quality of the action of the individual based on rules. From a deontological perspective what makes an action right is conformity to a moral norm and a major underpinning perspective is that no matter how morally good some consequences turn out to be, the choices that lead up to those good outcomes may be morally forbidden (Kant, 2011). Consequentialism on the other hand or consequentialist ethics focuses on the outcomes of the actions of a moral agent. Due to the fact that in consequentialism outcomes are usually all that matters the moral agent must act in the best way that achieves the best measurable outcomes (Bentham et al., 1996; Mill, 2001; Card and Smith, 2020). Virtue ethics focuses on the character of an individual and alienates the duties or consequences of actions by a moral agent. It focuses on the virtues one should possess and what actions are good or bad and how actions should be modeled after such behavior (Jantavongso and Fusiripong, 2021). Social Contract theory which is usually attributed to the works of Thomas Hobbes, John Locke, Jean-Jacques Rousseau, and John Rawls focuses on the social agreements that are established to allow individuals coexist in a society (Boucher and Kelly, 2004; Nimbalkar, 2011).

Based on the theoretical tradition in data ethics, the general choices made by a moral agent is usually centered on these traditional theories as the dominant approaches as they capture much of our moral intuition. This is not to say there are not a wealth of ethical theories that exist in the landscape of data ethics that can provide instruments for ethical analysis but many of them combine the dominant theories above to address several weaknesses (Ross, 1930; Stahl, 2012).

#### 2.3.3. Ethical theories meet data governance

**Figure 2:**
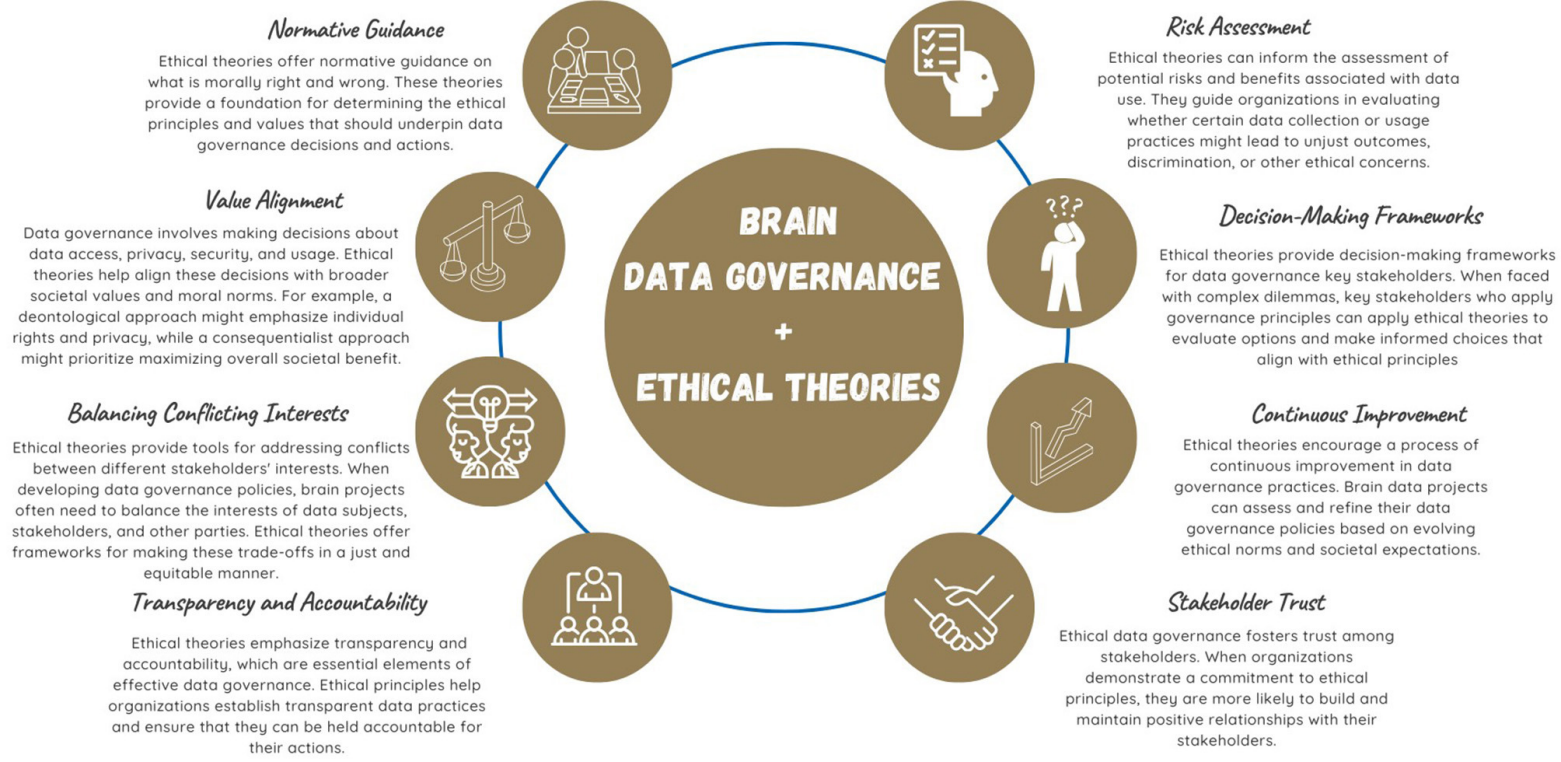
Ethical theories and brain data governance relationship.

The interplay between ethical theories and data governance is crucial in guiding how key stakeholders or brain research projects, and organisations collect, store, use, and share data responsibly and ethically. Ethical theories provide the philosophical frameworks and principles that help inform the development and implementation of data governance policies and practices. In Figure 2 we illustrate the relationship between ethical theories and data governance.

Ethical theories can provide the foundation for ethical decision-making within data governance. By integrating these theories into their practices, key stakeholders (e.g., researchers) and organisations who collect, store, and share brain data can ensure that their data handling processes are aligned with moral principles and societal values, ultimately leading to responsible and trustworthy data governance.

## 3. Research design

The research aims to investigate the ethical perspectives that form the basis of discussions on brain data governance. Uncovering the ethical considerations guiding the decisions, actions, or viewpoints of key brain data stakeholders can offer deeper insights into comprehending the diverse contextual foundations. This, in turn, can contribute to a better understanding of the broader landscape of global brain data governance.

### 3.1. Method

This study makes use of a secondary analysis (Creswell and Creswell, 2018) of interviews used for a previous study. The interviews involved 21 neuroscientists drafted from the International Brain Initiative (IBI), LATBrain Initiative and the Society of Neuroscientists of Africa (SONA) who are involved in various brain projects globally as shown in Table 1. The primary focus of the interviews was to find out the ethical and legal principles or issues that could arise in brain data research. The previous study focused on understanding the practical experiences relating to the influence ethical and legal principles had on brain data governance. The primary analysis and results identified statements around practical ethical and legal principles and issues that could arise in brain data research which majorly circulated around human rights, research ethics, participatory governance, and policies, regulations, and guidelines.

**Table 1:** Demographics of neuroscientists in the study.

**Geographical distribution**	**Number of participants per region**	**Profession (position in neuroscience)**	**Gender**
Africa	3	13 Professors/researchers in senior research positions 8 Early career researchers	6 females 15 males
Latin America	3		
Europe	4		
North America	7		
Asia	3		
Australia	1		

The richness of the quality of data collected in our previous research informed this study. The fact that neuroscientists who were respondents expressed various ethical and moral dimensions around the application of ethical and legal principles and the issues that arise from brain research provided the need to carry out exploration to understand their ethical positions. Therefore, a secondary analysis of the primary data was carried out to identify interesting findings that are independent of the original research. For data collection, we used neuroscientists as key stakeholders compared to other stakeholders such as policymakers and research participants because neuroscientists involved in brain data research are faced with different ethical decisions which puts their moral compass into question through practical decision making. Therefore, neuroscientists are at the forefront of the application of ethical and legal principles in brain data research.

Transcripts derived from the interviews were analyzed using NVivo qualitative analysis software (QSR International, 2021). The transcripts were read in their entirety to understand the meaning of sentences and phrases through which there was an expression of ethical values relating to an ethical principle. Then thematic analysis (Kiger and Varpio, 2020) was used to categorise the meaning of statements under underpinning theories that could be used to explain the underlying statements containing an ethical principle. Although subjective interpretations were used to analyse the statements, standardisation of the analysis was achieved through consistency checks and a process of deliberative mutual adjustment known as reflective equilibrium (de Maagt, 2017).

## 4. Results

The analysis provided results which describe the theoretical underpinnings around the statements of the respondents. Through the analysis the ethical positions of the neuroscientists are presented below in the form of deontological, consequentialist, virtue and social contract positions.

### 4.1. Deontological positions

The participants emphasised on certain duties and rules which should be applied toward data subjects and brain data. Participants also expressed rights and duties irrespective of the consequences.

Participants emphasised on the need to maintain privacy, protection and confidentiality of both research subjects and their data because they see this as an obligation and a core issue in brain data research especially when sharing data. Respondents also believe Ethics Research Boards (ERBs) are heavily influenced by the need to maintain privacy and this is prioritised and fundamental to ethics approval. Although participants see maintaining privacy, confidentiality, and the protection of participants as an obligation, there is a lack of agreement on best practices as different words are used to quantify maintenance of privacy and confidentiality. For example, participants used words like “blinding”, “coded” or “defaced” or “de-identified”. Participants also have a perception that they have a duty or obligation to inform research subjects when incidental or abnormal findings occur in brain research, however maintaining the need for privacy and confidentiality often conflicts with such obligations.

With regards to consent the participants expressed their obligations regarding processing consent as a rule for the collection of brain data. Participants also expressed the inadequacies of current consent models which calls for extra obligations. For example, a participant emphasised that the obligation to collect consent extends to providing communication and clarity to data subjects to understand the potential downstream uses of data that might not be anticipated as at the time of collecting consent. Also, in the use of invasive technologies there is a perception that the duty to collect consent should dynamically occur during the entire process due to the ability of deep brain simulations and invasive technologies to alter a subject's brain chemistry.

“*I attended this one seminar recently where an individual had Parkinson's and had this, like life saving deep brain stimulation technique that in 10 years, he would might have to get removed or, you know depending on how the trial goes, and someone brought up the question of, are you the same person as when you first signed your informed consent to 10 years later when your entire brain chemistry has changed? And at that point in time, you know, who does the informed consent lie on? You know, things change over 10 years when you're receiving this really invasive technology”. (North America 4)*

The respondents also raised deontological perceptions around engagement and the need to acquire brain data to carry out research. This necessity was in relation to the duty to educate data subjects on the need to acquire brain data to create interventions around brain diseases. One participant highlighted the stigma associated with brain diseases as compared to other diseases and stressed the importance of engaging and educating the society on the need to acquire brain data which might change the perceptions of data subjects to easily provide brain data for research.

“*because there's a stigma around that, the data is not readily available and presented……..you have to let people understand from an educational point of view, and the fact that we need this data, the fact that we can intervene”. (Africa 1)*

Respondents expressed the need for data subjects to have control over the decision-making process around their data. Words such as “free will” and “human rights” were used in reference to independence and autonomy. Some respondents expressed the need for large corporations to express more ways for data subjects to have control over their data.

Deontological views about fairness and transparency were expressed in relation to providing fair and equitable access to data which involved setting up requirements to allow data subjects to access and use their data. Also, respondents expressed concerns on how to make research results and the benefits of research available in a simple and understandable format to data subjects who make brain data available both for research and for the development of neurotechnologies. These concerns are driven by a sense of duty to promote accountability and some of the respondents pointed out that data subjects might be hit by paywalls when they attempt to access research outputs.

Perceptions around integrity were expressed around the rules and duties of data repositories and researchers. Views on data quality, open access and meeting FAIR (Findable, Accessible, Interoperable and Reusable) requirements were expressed with some participants raising arguments on how some datasets fulfill journal requirements but lack reusability.

“So *what I often see when they say, yes, all the data is on our website and open access. And then you see the kind of data they put are basically, they are unusable. But they fulfill the formal requirements for open access for the journal, but when you actually use it, forget it. You can't use it. And we had some big names there”. (North America 7)*

These views about the duty and rules regarding data integrity were further expressed by another participant with regards to brain data repositories who stated that the duty of ensuring that data is ethically acquired and meets FAIR rules is reflected in the open data commons of the repository and this basically outlines the liability of the data submitter. Therefore, the liability to ensure that data meets ethical requirements is shifted to the data submitter rather than those in charge of the repository.

Some participants emphasised on the need to protect their intellectual property (IP) as their perceptions illustrate their views on ownership. The perceptions of some participants show that there is currently a need on how best to acknowledge the owners of research outputs which is seen as a duty. There is also a perception that the ways of acknowledging people for their research outputs has evolved significantly but more strategies might need to be developed as one respondent pointed out that copyrights are the only way to protect intellectual output while the other stated that there are issues in acknowledging ownership.

“*They're kind of broader issues like provenance and how do you cite the people who did all the work to get the data and licensing issues and things like that”. (North America 6)*

“*the only thing we know is to protect your property you get a copyright”. (Africa 2)*

Some perceptions regarding the responsibility and accountability of parties involved in data submission were made in reference to the liability of repositories. It appears that there is a need to provide more clarity around the liability of improperly acquired data residing in repositories as one participant pointed that in most cases improperly acquired data is usually taken down without holding repositories liable. These perceptions hold some deontological views as to the duties of repositories in ensuring that data is properly acquired and used in accordance with guidelines.

“*I have become a little bit more concerned about repositories and their liability for breaches, right? Thus far, nobody has held the repository liable for data that was improperly acquired, the repository is expected to take it down”. (North America 3)*

With regards to the legal basis for the collection, processing, storage and sharing of brain data, while some participants explicitly mentioned laws that underpin their activities around brain data research, some participants pointed out the lack of laws and the non-clarity of guidelines, policies, and procedures. This non clarity results in conflicting views and sometimes lack of guidance on the rules and duties in brain data research from a deontological perspective.

“*we want to you know, stick to the regulation and the law, but the regulation and the law is not completely clear. So, what is personal information is very straightforward in some field, like legal fields, but in neuroscience data, it is kind of difficult to define what is personal information, because we are dealing with brain imaging data of a person or a patient”. (Asia 3)*

Most of the respondents expressed the need to prevent harm and to promote the welfare of data subjects and research participants. This is also included situations where incidental findings occur and one participant pointed out that researchers have an obligation to pass the data of subjects to trained clinicians when incidental findings surface. The use of fundamental human rights was acknowledged by participants as a good approach to promote beneficence and non-maleficence. However, one respondent emphasised that while researchers are obliged to protect and promote the welfare of participants, current regulations that criminalise brain diseases and mental health may cause harm to data subjects.

Deontological views expressed around the retention and destruction of brain which involves complying with data management procedures shows that the respondents adopt different rules in the retention and deletion of brain data. Some of the respondents agree that destruction of data is the right of a data subject. However, the perceptions of the various respondents also show that there are major divisions in the arguments of the respondents around if brain data should be destroyed and when it should be destroyed or if it should be retained continuously.

“*So having a little bit of standard, like, keep your data at least for 10 years so that people can reproduce your results”. (Europe 3)*

“*I don't think data over ten years can be necessarily helpful or useful for research anymore because of the advancement in research”. (North America 5)*

“*the better way to destroy this data is to, but this is very, very tricky, is to confirm to be very, very sure that the anonymization of the data is being done properly”. (Latin America 1)*

### 4.2. Consequentialist positions

Participants emphasise on the consequences of not having appropriate measures to ensure privacy, confidentiality and data protection when sharing brain data. Some of these consequentialist arguments occur especially during the sharing of brain data and in the form of brain data images as there is a perception that current privacy, confidentiality, and protection techniques do not fully address re-identification. For example, in terms of personalised medicine through modeling, a respondent had a perception that participants can be identified because human imaging data is used to build models. Also, the misuse of brain data is considered as one of the most feared points by neuroscientists as pointed out by a respondent. The potential for misuse is also expressed in terms of the reuse of brain data. Although the reuse of brain data can provide valuable insights when combined with other data to form big data a consequentialist argument raised by one of the respondent points to the fact that it jeopardises the privacy of the individual especially with regards to broad reuse.

“*there's a real tension between very broad reuse, and having approval for broad reuse, where you can ask many possible questions or you can link the data with other data, which is a major challenge, because potentially it jeopardizes the privacy of the individual and it also alters the types of questions that can be answered”. (North America 2)*

This perception relates to another consequentialist argument about privacy, confidentiality and protection which is based on the lack of definition of what is classified as personal data or personal information which comes from the perception that the shape of the brain could be a fingerprint use to identify an individual from their brain imaging data.

“*Yeah, and there is no restriction of the sharing of brain image data in terms of personality you know as personal information…. one possibility is that the shape of the brain could be a kind of personal information, because as you know, the brain has a very complex shape different people have different gyral patterns or sulcal patterns. So that is kind of a fingerprint. So, its maybe possible to identify a person from his MRI data that is possible”. (Asia 2)*

Consequentialist arguments raised by participants around consent suggests that consent is a tool for research subjects to control their data and this control leads to trade-off between privacy and data utility. According to participants consent should be limited by law as data subjects can never fully understand the implications of consent around data sharing.

“*I don't think it's entirely possible, I think people can't completely understand what could be the implications of sharing, and that this consent should also be limited by law. So, it shouldn't be possible to consent to anything”. (North America 1)*

This argument is reflected by another participant who pointed out that consent limits data utility especially when sharing data across borders because consent forms cannot be modified during cross border sharing.

Perceptions around bias and discrimination expressed consequentialist views of using biased brain data samples for the development of algorithms. Some participants argued that although bias in brain data and algorithms can be unconscious or unintentional this generates risks, and these risks will always surface regarding the testability and reliability of algorithmic decisions. Some participants also believe that US and EU regions will have to deal with more issues of bias and discrimination due to the prominent level of ethnic diversity.

Respondents also raised views around engagement which centers on ERBs. Some respondents pointed out that ERBs which are supposed to review and approve research to ensure compliance usually lack the necessary education and expertise to understand data related issues and concepts which results in barriers in brain data research. Therefore, more engagement and education of ERBs might be needed to provide exposure to some of the data related concepts as a lack of these expertise might prevent fairness in the ethics review process. This is also stressed by another participant who pointed out that while ERBs are necessary there is currently a lack of sufficient competence in turning complex legal and ethical frameworks into practice therefore resulting in consequences that affect research approval.

“*And in general, you know, what is a big big issue is that these ethics review boards, and there's called different things, IRB, or REBs, etc. But these are, very rarely actually have the education necessary to understand, for example, the real issues around the data and the data use and reuse. And so I think there's a critical need for education of these ethics review boards, to understand that you know, data and data related issues”*. *(North America 2)*

Consequentialist views around autonomy and independence pointed to the need to structure ERBs which are free from institutional control especially in the process of ethics approval. Participants emphasised that independent ERBs free from institutional control are important as a lack of such independent and autonomous ERBs can stall research projects. A practical example was illustrated by a participant who narrated how ethics approval for a project was stalled due to the responsible institution proceeding on strike due to an industrial dispute.

“*You could submit an approval today, tomorrow, you come back and hear that the teaching hospital is on strike and everywhere is shut down. There is nothing you can do about it and it can take one full year and you can't do anything about it, your research will suffer”. (Africa 3)*

The consequentialist views around fairness and transparency focused on the lack of procedural fairness in ethics approval by ERBs. There is also a perception by some respondents that ethics approval are control instruments and create a sense of lack of procedural fairness due to the evolving and complex nature of ethics approval. For example, a participant highlighted an example where the use of the word drone required further clarification and justification for the research while the use of the word robot was seen as less sensitive.

Perceptions regarding data integrity were expressed in terms of open access and requirements for FAIR. The underpinning consequentialist view as portrayed by a respondent is that while public funding bodies, data repositories and publishers promote open access, there is currently a non-democratic effect regarding open access especially in regions with little or low access to funding. This is because the structure of open access usually requires payments that might stall open access and sharing in regions with low research funding.

“*open access was supposed to be a tool for everybody in a more democratic way for everybody to have access to science, but then it starts to become a business…… I have seen a lot of those examples and that's very bad and that is also something that affect directly countries where we don't have good funding”. (Latin America 3)*

Although the consequentialist views on the legal basis for processing brain data focused on the complications that arises in brain data sharing among jurisdictions, participants also placed emphasis on the lack of regulations tailored toward brain data which might result in unintended violation of procedures and guidelines. This lack of regulations was mostly highlighted by participants in the African and Latin American region and one participant pointed out that the lack of brain data regulations can result in misuse. while another participant from the Asian region referenced the GDPR (General Data Protection Regulation) stating that it provides clarity and prevents such unintended consequences in the management of data.

“*But I do say that I respect the procedure of the EU that they have or they clearly state some global law for the whole of Europe and then get consensus and then researchers try to follow such a global law. This is very important for helping not only the people but also the researchers. We don't need to think too much”. (Asia 3)*

Respondents also expressed views on the balancing of risks and the benefits of research by ERBs which can be considered as the principle of proportionality. One respondent explained that while there are varying ethical and legal principles which creates challenges in the analysis of risks by ERBs, there has been too much emphasis on the risks of brain data research rather than the benefits. These risks mainly focus on data reuse and data linkage therefore creating a lack of proportionality in the ethics approval process by ERBs.

“*The other aspects that I think is so, so critical, is, there's so much emphasis on risk in a negative sense, right, and the risk of data sharing and data reuse and data linkage. It's the big focus for many of these ethics boards is on risk”*. *(NorthAmerica2)*

Strong consequentialist views were expressed with regards to neurorights. Although many of the respondents had not heard of neurorights, it appears that some neuroscientists believe that neurorights will promote transparency, openness, protection, and privacy which results in the overall good of data subjects and the responsible use of neurotechnologies. Also, respondents argue that due to the rapid evolution of neurotechnologies such as brain computer interfaces and neuronal implants, neurorights might assist in curbing both intentional and unintentional consequences of such technologies. Although some respondents believe that neurorights might have positive consequences some also expressed views that shows that neuroscientists believe that neurorights might create additional hurdles in navigating the already complex set of ethical and legal guidelines. Some other arguments point to the fact neurorights might stifle the development of neurotechnologies and advancement in neuroscience research. One clear example was raised by one of the respondents who expressed persuasive arguments by pointing out that search engines and social media modulate the brain in a more efficacy way than neurotechnologies and questioned why special rights have not been proposed in such areas.

“*do you realize how much your search questions to Google actually tell you about your brain? and how much social media actually modulates your brain in a far more efficacy way and in a far better understood way, than any neurotechnology today? And do we have anything controlling that? Do we have any rights there? No, not really”. (North America 2)*

The consequentialist views about the destruction and retention of data as a principle was expressed especially around the need to retain data without destruction and the consequences of the deletion of data which reduces the greater good in brain data research. For example, a respondent pointed out that in terms of reproducing brain data research findings, the ability to combine multiple datasets to achieve big brain data can provide neuroscientists with valuable insights which will be beneficial to the society. Therefore, the constant deletion of data prevents such achievements. This is also expressed by another respondent who stated that deletion of data might violate the rights of data subjects and society to a good healthcare system because AI can be used with such large datasets.

“*the rights of society for a good health system and the rights of patients to get the best possible treatment, which especially if we talk about new health applications that are based on AI, for example, require the availability of large amounts of health data”. (Europe 4)*

### 4.3. Virtue positions

Respondents emphasised on the need for neuroscientists to express attributes that promote research integrity especially with regards to open access and data sharing. There is a perception that researchers are usually unwilling to share data due to the need for competitive advantage. However, a participant pointed out that developing regions tend to display more attributes with regards to open data sharing as it might be a prerequisite to gaining access to data.

Responsibility and accountability were expressed as professional characteristics that neuroscientists involved in brain data should possess. These were expressed in different dimensions around the sharing of data and around the development of Artificial Intelligence (AI) using algorithms derived from brain data. One respondent pointed out that the responsibility of brain data researchers does not end when data leaves a jurisdiction because poorly acquired data might have strong ethical and legal implications. Also, there were strong views on the responsibility and accountability of neuroscientists involved in the development of AI under government frameworks. These views focused on the need to express responsibility in defining the interaction and relationship that is established between end users of AI which might also include animals with some highlighting the need to have reflexivity when developing AI for militarisation as operating under a government framework may be legal but unethical.

“*we will have to take into account that these autonomous machines will interact with people….it could be also animals….. if you programme a robot to do some military stuff, and it could be okay legally, because you are on a government framework, but it cannot be ethical in terms of your profession”. (Latin America 3)*

### 4.4. Social contract positions

With regards to the privacy of data there is a perception that there needs to be an agreement regarding the level of privacy, protection, and security of data in infrastructures. This view comes from a need to balance privacy and utility of data as sometimes the request to implement privacy, security, and data protection procedures in infrastructures reduces the usefulness of data. Also there appears to be a need to have an agreement on the sharing of face data with human imaging data as these considerations will increase privacy and confidentiality and reduce the risk of re-identification.

“*The apparent concern still, is if we can share people's face, which is often included in human imaging data, and we should remove his or her face from MRI data that is there is an active discussion going on and theoretically, we should remove face data from the MRI data that is almost established as a fact and now we need to follow that idea”. (Asia 2)*

The social contract theory underpins consent based on the need to develop or agree on appropriate consent models as respondents pointed out that current consent models cannot handle the peculiarities of brain data research. Participants appear to be highly influenced by the need to carefully manage consent during data sharing due to different translations of consent and the nature of brain data.

Using the focal lens of the social contract theory shows that some participants expressed the need for common ground of rules regarding the oversight applied to the research community and to the private sector around data collection and data sharing. This is expressed in the form of the research community having a closed market approach while the industry has a free markets approach when it comes to data sharing. Therefore, having a common ground of rules will reduce a sense of discrimination while promoting equity and fairness.

“*I think we should have a common ground rule of ethical principles that would apply to both researchers and in the industry. What can a researcher do in terms of data, collection and sharing shouldn't be different from what a company can do with data collection and sharing”. (North America 5)*

Respondents emphasised on the need for more engagement regarding the change of certain existing legal frameworks that criminalise brain data diseases and mental health. For example, a participant pointed out that some countries currently have laws which portray attempted suicide as a criminal offense whereas attempted suicide might be related to a brain condition. As a result of this, individuals with brain related conditions are treated as criminals.

Using the focal lens of the social contract theory, perceptions were deduced regarding the validation of data generated by simulators in the field of brain data research to promote data integrity. Based on this view, neuroscientists are influenced by the need to develop rules and agreements on how to measure and validate the correctness of simulated brain data. This comes from the view that experimental data might be easily validated but they appear to be limited ways to measure simulated brain data.

“*of course, with simulation data, the correctness of the data itself is harder to validate”. (Europe 1)*

“*How do we check or how do we guarantee this data generated from simulators are okay?”. (Latin America 1)*

Some perceptions regarding data ownership highlight the necessity for a common set of rules concerning the open sharing of data to align with FAIR principles and meet the requirements of funding agencies. Currently, there exists tension in balancing the need for open access and the expectations of funding agencies, which may be the owners of the research data. This is in relation to the need to develop laws that can protect ownership of brain data especially in repositories as stated by a respondent pointed to the fact that current licensing structures in repositories do not provide appropriate mechanisms for apprehending persons who or abuse or misuse data.

“*We don't have an apparatus to go after people who are illegally using our data. So we may as well just give it to them, because we're not going to go after them and we know we're not going to go after them, right. And I think that those legal issues are really important because the repositories that acquire this data, and that's always where I come from, are not in a position to do this”. (North America 3)*

Strong emphasis was placed around developing a common structure for handling ethical and legal compliance in the academic and private sectors. This perception comes from providing clarity around the legal basis for processing brain data and participants pointed out that in academic research the collection and processing of brain data is well regulated with visible structures such as ethics review boards as compared to the private sector which has more brain data in its possession.

Respondents expressed perceptions around trust which majorly focused on the protection of intellectual property and reciprocity in brain data sharing. One respondent pointed out that there is a certain level of suspicion which accompanies data from other regions majorly because there is a lack of knowledge of the ethical and legal procedures used in obtaining brain data in other regions. Therefore, this lack of knowledge might also be accompanied by a lack of guidance on ethical and legal equivalence between two data brain sharing parties in different regions.

“*we are more suspicious of data that is submitted from other countries than our own, simply because we understand the rules in our own country, and we don't understand them and the others, and we don't have the staff to go and say, oh, yeah, no, this is equivalent and this is okay. But I think that sort of guidance is a problem”. (North America 3)*

Although there were perceptions on the consequences of the adoption of neuororights, there was a high level of agreement that further discussions around the conceptualisation of neurorights is required. Assumptions and suggestions were made on what neurorights should entail and guiding questions were also proposed by some respondents. Some of the suggestions involved engaging the wider community and different stakeholders in the conceptualisation of neurorights. Questions such as what is the scope of neurorights? does consciousness come into play? is it only for research or the private sector? were also raised by some respondents which shows the need for clarity and agreements.

“*I'm curious to see what it entails. Right? It seems a bit more off the top of my head…..Like, should everyone have access to their own thoughts? What does that mean? What's a thought? Right? does consciousness come into play? Should we have something about the untouchable rights to an identity? What is an identity?”. (Europe 2)*

Although the respondents expressed various perceptions which underline the fact that they operate under different brain data retention and destruction guidelines, there were also expressions which showed that there is a lack of clarity on the guidelines around data retention and destruction. Some of the participants provided various suggestions on how to provide clarity in such guidelines. One participant suggested that to provide such clarity principles investigators, laboratories, funders, and journals need to come to agreement with regards to the criteria for data retention and destruction.

## 5. Discussion

**Figure 3:**
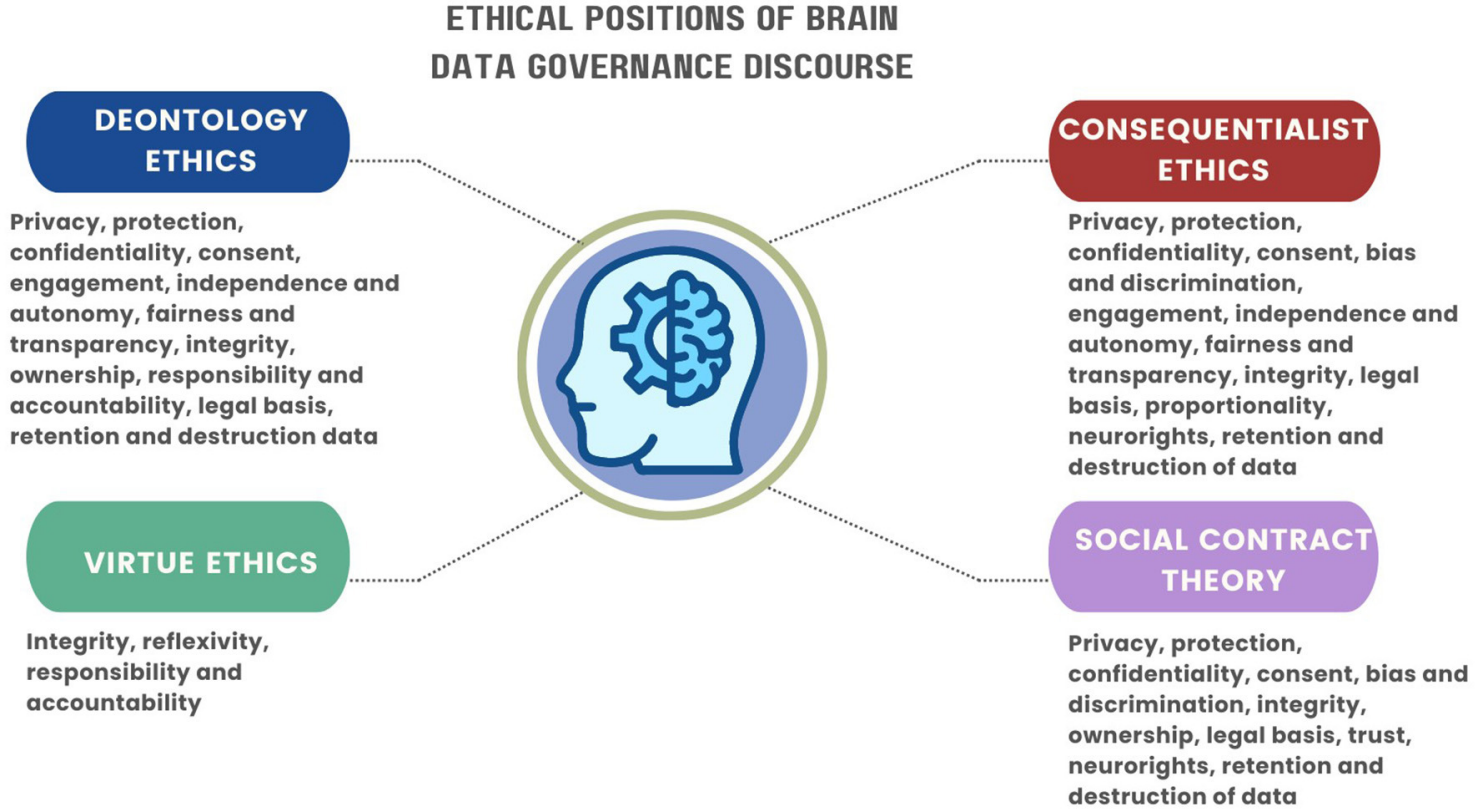
Ethical and legal principles and their underpinning ethical positions.

The ethical analysis above presents various ethical and legal principles reflected in the discourse and the underpinning ethical positions. These discussions reflect the contextual perceptions in the conduct of brain data research and the management of brain data. In Figure 3 we present an analytical illustration of the various principles in the discussions and how they are underpinned by various ethical positions.

### 5.1. Summary of insights and critical recommendations

In Table 2 we offer a condensed overview of the observations derived from the contextual stances and perspectives of the neuroscientists involved in this study.

**Table 2:** Summary of key insights.

**Deontology positions**	**Consequentialist positions**	**Social contract positions**	**Virtue positions**
*Privacy, Protection, and Confidentiality*: Insights show an emphasis on the obligation to maintain privacy, protection, and confidentiality of research subjects and their data in brain data research, especially during brain data sharing. However, there is a lack of agreement on best practices, with different terms like “blinding,” “coded,” or “de-identified” used to quantify such obligations which calls for standardisation.	*Consent and Data Utility*: Key insights around consent suggests that consent is a tool for research subjects to control their data and this control sometimes lead to trade-offs between privacy and data utility especially when sharing brain data across borders where consent forms cannot be modified. Some insights suggest consent should be limited by law as data subjects can never fully understand the implications of consent around data sharing.	*Balancing Privacy and Data Utility*: Participants emphasise the need for an agreement on the level of privacy, protection, and security of data in infrastructures while considering the balance between privacy and data utility. This insight highlights the importance of implementing privacy and security measures without compromising the usefulness of data.	*Ethical Considerations in AI Algorithm Development*: Virtue positions provide ethical considerations surrounding the development of AI algorithms that utilise brain data under different legal frameworks. The insight indicates that researchers and developers need to be mindful of the ethical implications of AI algorithm development, even if certain practices are legally permissible.
*Consent and Communication*: Participants recognise the duty to process consent for the collection of brain data. They express concerns about the inadequacies of current consent models, calling for extra obligations. Clear communication with data subjects is highlighted, ensuring they understand the potential downstream uses of their data, especially when using invasive technologies that can alter brain chemistry of brain data subjects.	*Appropriate Privacy and Protection Measures*: Participants emphasise the need for appropriate measures to ensure privacy, confidentiality, and data protection while sharing brain data especially in the form of brain data images. The insights obtained show that current techniques may not fully address re-identification risks, especially in the sharing of brain data images and in personalised medicine modeling due to the use of human imaging data to build models. This can also be linked to the lack of clarity in what can be classified as personal information in the context of brain data.	*Development of Appropriate Consent Models*: The perception by participants underscores the practical need to develop appropriate consent models in brain data research, as current models may not address the unique aspects of brain data. Along with development of appropriate consent models insights also emphasise the significance of carefully managing consent during data sharing to ensure ethical practices.	*Importance of Ethical Virtues*: Insights show that key stakeholders in brain data research should possess certain ethical virtues, including integrity, reflexivity, responsibility, and accountability. This insight emphasises the practical significance of these virtues in guiding ethical decision-making and behavior in the context of brain data research.
*Citizen Engagement and Education*: Key insights obtained emphasise the duty to engage and educate data subjects about the necessity of acquiring brain data for research, especially in creating interventions around brain diseases. Addressing the stigma associated with brain diseases is crucial in changing perceptions and encouraging data subjects to willingly provide brain data for research.	*Education and Independence of Review Ethics Boards (REBs)*: The insights highlight the importance of engaging and educating ERBs to understand data-related issues and concepts and lack of expertise in these areas may hinder fairness in the ethics review process. Practical insights also stress the need for independent REBs free from institutional control to ensure efficient ethics approval as the lack of autonomous REBs can delay research projects, affecting their progress.	*Establishing Common Ground Rules for Research and Industry Oversight*: Insights point toward the need for common rules and agreements regarding data collection, processing and sharing practices between the research community and the industry or private sector. Such common ground would promote equity and fairness in practices involving brain data. This insight also highlights the importance of clarity in processing brain data within different sectors.	*Necessity of Responsible Research Conduct*: Insights about the necessity of responsible and ethical conduct in brain data research are also deduced. It underscores the importance of adhering to ethical principles, being accountable for research actions, and maintaining transparency in data sharing, all of which are practical measures to ensure the ethical progression of brain data research.
*Data Subjects' Control*: Theoretical and practical insights stress the importance of data subjects having control over their data, promoting independence and autonomy. The need for large corporations to offer more control over data is emphasised, as data subjects should not be treated as a means to an end but as autonomous individuals. Therefore, this calls for more frameworks that permit citizens or data subjects to have access and control to their brain data which is essential in the governance of brain data.	*Open Access and Data Integrity*: While public funding bodies, data repositories and publishers promote open access and although open access is considered to advance data sharing participants express concerns about the non-democratic effect of open access, particularly in regions with limited research funding. Key insights indicate that payment requirements for open access may hinder data sharing in such regions which can affect data integrity and open access goals.	*Revising Legal Frameworks that criminalise brain diseases*: Participants express perceptions on the need for more engagement to change existing legal frameworks that criminalise certain brain data-related conditions. This insight highlights the necessity of revising laws to provide adequate care for individuals with brain conditions rather than criminalising them which can promote the welfare of brain data research participants and promote human rights.	
*Fair and Transparent Access to Brain Data Research Outputs*: Deontological views highlight the duty to provide fair and equitable access to data, allowing data subjects to access and use their data. Ensuring research results and benefits are accessible in a simple and understandable format is crucial to promote accountability and avoid hindrances like paywalls for data subjects trying to access research outputs. This also promotes participatory brain data governance.	*Misuse of Brain Data*: The potential misuse of brain data is a major concern among neuroscientists. The reuse of brain data, while offering valuable insights when combined with other data, is viewed as potentially jeopardising individual privacy, especially in cases of broad reuse.	*Validating Simulated Brain Data*: The insights show that neuroscientists emphasise the need to develop rules and agreements to validate the correctness of simulated brain data, as it may lack standardised measurement methods compared to other forms of brain data.	
*Brain Data Repositories and Data Integrity*: Perceptions about data integrity revolve around the rules and duties of data repositories and researchers. Participants raise arguments on data quality, open access, and meeting FAIR requirements. Ensuring that data is ethically acquired and meets FAIR rules is considered as a shared duty, shifting the liability to data submitters to ensure ethical compliance. However, there is also a need to clarify such liabilities in the use of compliance structures such as data use agreements (DUAs).	*Proportionality in Ethics Review*: Insights show the need for a balanced approach in ethics approval, considering both the risks and benefits of brain data research. Key insights suggest that the focus should not solely be on risks but also on the benefits of research and involving key stakeholders such as research subjects who have experienced brain related conditions can be used to provide fair evaluations during such review.	*Establishing Common Agreements and Guidelines for Data Ownership and Sharing*: This position points to the need for a common ground set of rules for open data sharing, meeting FAIR rules and funding agency requirements while protecting data ownership and intellectual property. Practical insights also highlight the importance of developing laws and guidelines to protect brain data ownership in repositories. This might also involve the restructuring of licenses used in repositories and developing appropriate technical safeguards.	
	*Neurorights Perspectives*: Insights show that some neuroscientists believe that neurorights can promote transparency, protection, and responsible use of neurotechnologies, while others see neuroright as potentially hindering research and development.	*Building Trust in Data Sharing*: The insights highlight the need for trust in data sharing, especially concerning the protection of intellectual property and reciprocity. Participants emphasise the necessity of transparent ethical and legal procedures in obtaining and sharing brain data from different regions.	
	*Data Destruction and Retention*: Insights obtained from the ethical positions highlights the consequences of constant data deletion, which may hinder valuable insights and advancements in brain data research. Therefore, retaining data can support reproducibility and benefit society. Retained data allows combining multiple datasets to achieve valuable insights, benefiting society and healthcare systems that leverage AI with large brain datasets.	*Conceptualising Neuororights*: The practical need for further discussions and agreements on the conceptualisation of neurorights are also provided. Practical insights suggest engaging various stakeholders in the process to define the scope and guiding principles of neurorights. Insights also show the gaps in conceptualisation of neurorights as key figures in brain data research are not aware of the concept and are not involved in discussions.	
		*Providing Clarity in Data Retention and Destruction*: Practical insights emphasise the importance of establishing clear guidelines for data retention and destruction. Participants propose the involvement of investigators, laboratories, funders, and journals in reaching agreements on such criteria.	

Table 2 below also reveal key insights that need to be considered in facilitating the development of a global framework that encompasses contextual considerations. The study reveals the underlying tensions and internal contradictions when considering current ethical and legal frameworks and how they influence the conduct of neuroscience. Some of the views of the respondents reflect concerns, issues and possible recommendations which can serve as catalyst for the understanding current contextual challenges in brain data research. When considering the views of the respondents it can be observed that there are currently internal contradictions and tensions between the need to advance neuroscience, the rights of data subjects, the obligations of neuroscientists, current structure of ERBs and data repositories.

It appears that the need to maintain privacy, confidentiality, and protection sometimes conflict with the need to share brain data as current safeguards might be considered inadequate in handling issues that surface during sharing and processing. For example, a participant expressed the possibility of reidentification even with the use of anonymisation during sharing. This is in relation to several practices used in enhancing privacy and data protection. Certain agreements need to be established in determining if a blinded, coded or pseudonymised data is equivalent to an anonymised data. Also, these agreements have to be in conjunction with an underpinning law overseeing such privacy enhancing techniques in the relevant context. Such agreements might need to go in tandem with a global consensus around situations such as the sharing or removal of face data in human imaging data and what constitutes personal information. This is in line with the argument raised by a respondent asserting that the shape of the brain can be classified as personal information with people have different sulcal and gyral patterns which can serve as fingerprints. This is supported by the GDPR (Feiler et al., 2018) which states that if an individual can be identified from the information being processed then such information can be classified as personal information. Such arguments show that different context gives rise to different expectations and preferences in relation to data protection, privacy, and confidentiality (Nissenbaum, 2004). Furthermore, while the deontological view of protecting the data of a data subject is essential as prescribed by several laws and guidelines, consequentialist views raise tensions around the utility of brain data when advanced privacy enhancing techniques are applied without recognising the need to enhance utility. Therefore, the importance of creating a balance between the utility of data while increasing access is essential (Eke D. et al., 2021) as reduction in the utility of data reduces the overall benefit of brain data as a result of overprotection which can indirectly reduce the benefit of such data to science and society.

The need to create such balance between the rights of data subjects and the rights of the society to benefit from brain data research is also reflected in the broad reuse of brain data. Such practice involves combining multiple brain datasets to achieve big brain data which provides valuable insights. Although respondents provided views which promote broad reuse of data, consequentialist concerns enable us to identify the risks involved with broad reuse in two dimensions. First is the risk of reidentification which involves jeopardising the privacy of the data subject and secondly the risk of voiding consent because broad reuse alters the original questions in the design of the research and consent. With the advent of commercial actors and interest in AI, these risks will tend to increase as such AI technologies or neurotechnologies rely on substantial amounts of brain data for the training of datasets which are used to develop such technologies. This is in line with deontological views on the inadequacies of current consent models in handling brain data research and the call for better consent models (Ochang et al., 2022; Wiertz and Boldt, 2022). However, the consequentialist view raises internal contradictions around consent which raises tensions with broad reuse. For example, one of the respondents pointed to the fact that research participants do not usually understand the implications of consent and therefore consent should be limited by law. This shows that some researchers see consent as a tool used by data subjects to control their data and minimise risks which reduces the ability to share data and to use data for broad reuse. This can be pictured as causing harm indirectly because it prevents the ability to utilise brain data to the benefit of society and sometimes the data subject.

Therefore, the role of ERBs involves analyzing brain research projects and balancing such risks during approval in other to enhance proportionality as pointed out by some of the views. This involves having the necessary expertise in review boards to carry out such reviews without placing a necessary emphasis on risks in a negative sense. Some of the views point to the fact that ERBs do not have necessary expertise to carry out such balancing of risks as there is currently a lack of knowledge in data related issues and the converting of complex ethical and legal frameworks into practice. A typical example expressed by one of the respondents was that there is the concept of a GUID (globally unique Identifier) used by the NIH to make a linkage between studies where the same individual participates in multiple studies. This makes it possible to create an anonymous identifier that allows the linkage of individuals across data without storing their personal health information or their personally identifying information. However, the respondent states that very many ERBs or reviewer boards are not familiar with a lot of these concepts. These raises perceptions and views around a lack of procedural fairness. Therefore, deontological, and consequentialist concerns provide views that shows the need to engage and educate ERBs which will ensure that decisions about risks are well informed.

This is also in relation to the concerns around the structure of review boards due to the evolving landscape of research and the need for alternate review board models (Grady, 2015). Consequentialist views point to the fact that review boards need to be independent and autonomous as a lack of these might stall research in situations where industrial disputes arise. Also having patients, family members, and people with lived experience, for example, involved in the governance of ERBs can also enable ERBs carry out a fair evaluation of brain data research projects during review as these are stakeholders who can make judgements and conclusions on the merits and demerits of brain data research based on past experiences. With commercial actors now becoming increasingly active in the use of brain data, the existence of commercial review boards (Lemmens and Freedman, 2000) as visible oversight structures might also be necessary in ensuring that brain data is used responsibly which might also promote trust. This is because the view by some of the respondents show that commercial actors have less visible oversight structures as compared to the research sector.

The deontological and consequentialist views also allowed us to gain insights into the perceptions around brain data repositories, intellectual property, data integrity and FAIR. Deontological concerns revealed the issues around the responsibilities of repositories in ensuring data integrity, provenance tracking and intellectual property. To promote FAIR, repositories use data use agreements (DUAs) (Eke D. et al., 2021) licenses such as CC0 or CC BY licenses as ethical and legal instruments to ensure that brain data is obtained and submitted ethically (Hrynaszkiewicz and Cockerill, 2012). DUAs also ensure compliance in repositories. The deontological views around the inability to understand how to enforce licenses and DUAs in repositories to carry out legal actions based on the misuse of brain data obtained from repositories or the submission of unethically acquired brain data shows that some of these instruments fall short in addressing issues of misuse as they do not provide strong mechanisms for enforcement. it also raises arguments around the need to promote open access and the need to promote data integrity. Although increasing procedural hurdles around repositories can be viewed as draconian due to the procedural challenges already encountered in the collection, sharing processing and storage of brain data, it calls for an understanding of the implications of putting these licenses and data use agreements in place and an understanding of how they are going to be enforced and if they are going to be enforced. This in line with the views of some of the respondents who pointed out that some of the data in repositories meet FAIR and open access requirements of journals but are unusable. Such views call for clear and visible structures which define liability and the development of visible global structures for the verification of ethics approval for brain research.

With the advancement of brain data research and the development of neurotechnologies, there have been recent discussions about neurorights which focuses on establishing guidelines around protecting human rights as neurotechnology advances. While they have been many arguments in literature, the ethical positions of respondents which are mainly consequentialist in nature adds to the current debate in the framing of neurorights (Ienca, 2021; Rommelfanger et al., 2022). There were mixed views regarding the framing of neurorights and most of the respondents had not heard of the concept of neurorights. This raises a fundamental concern in the framing of neurorights as the respondents who are neuroscientists involved in large scale projects had not heard of neurorights. The consequentialist view by the respondents also justifies some of the claims by previous research regarding the framing and conceptualisation of neurorights. The contextual views regarding what neurorights should entail provides important insights and shows that neuroscientists who are important stakeholders should be involved in the framing of such rights. For example, some of the views asked if thoughts, consciousness, and identity should also be considered and if there is a hard border between what is considered as “neuro” and the rest of the body. Also, the respondents have perceptions that neurorights will add to the already complex landscape of ethical and legal issues and might prevent the collection of brain data for research and the advancement of neurotechnology which can benefit the society and data subjects. Some of the respondents from the European region also pointed out that brain data is well regulated, and the rights of data subjects are well protected and such new rights will have more effect in other regions where regulations such as the GDPR and AI act are lacking.

This is in line with the deontological and consequentialist positions around the legal basis for the collection and processing of brain data. The deontological positions expressed the lack of clarity in the laws which results in the inability to follow rules and perform duties while the consequentialist views expressed the consequences of such inability to follow rules due to the non-clarity or nonexistence of brain data laws and guidelines. Some of the concerns show that in some regions existing ethical and legal frameworks in some regions do not provide the necessary guidance to ensure compliance when carrying out brain data research which results to both intentional and unintentional misuse of data and even violation of human rights. For example, some laws still criminalise attempted suicide which can be as result of a brain condition (Lew et al., 2022). Furthermore, the lack of clarity in legal basis can be observed around the contextual views relating to the retention and destruction of brain data as the different respondents propose different retention and destruction timelines for brain data.

In addition to deontological and consequentialist concerns the study also highlights virtue ethics which focuses on the moral virtues and traits that key actors should possess and express. Some of these include being accountable for data even after sharing, ensuring data integrity and quality, showing responsibility and accountability around the use of brain data for commercial AI and neurotechnologies. Some of the positions highlight the fact that researchers and other data users might sometimes prioritise the benefits of brain data research to society and scientific curiosity above the fundamental rights of both human and animal subjects. Sometimes this involves operating under a framework that is legal but unethical. Also, in the development of AI and neurotechnologies much emphasis has been placed on human subjects and their interaction with neurotechnologies but one of the concerns by the respondents highlights the need for also addressing the interaction of such neurotechnologies with animals which can violate animal rights. This is in line with arguments around the limitations of the 3Rs tenet, Replacement, Reduction, Refinement, and the call to embed responsibility and reflexivity in line with Responsible Research and Innovation (RRI) (McLeod, 2015).

In addition to deontological, consequentialist, and virtuous ethical positions the study also highlights the need for key actors to agree based on common interest which is underpinned by the social contract theory. The social contract theory aims to provide reasons why members of the society have reasons to endorse and comply with fundamental laws, institutions and principles based on a particular context (D'Agostino et al., 2021). Having a harmonised set of ethical and legal requirements for governing data ensures that neuroscientists and key actors involved in the management of brain data are acting ethically and are legally compliant. Based on the results the social contract theory encompasses the development of processes, guidelines and rules around several areas of concern such as balancing privacy and utility, agreements on the procedure for sharing brain images and definition of personal information in the context of brain data, development of consent models, common ground of rules regarding the oversight applied to the research community and to the commercial sector, decriminalising brain diseases, developing rules and agreements on how to measure and validate the correctness of simulated brain data, developing set of rules regarding the open sharing of data to meet FAIR rules while meeting the requirements of funding agencies and institutions, refining licensing structures in repositories to clarify liability and enforcement, developing a common structure for handling ethical and legal compliance in the academic and private sectors, developing sharing models around trust, agreements around the conceptualisation of neurorights, and agreement on data retention and destruction guidelines.

## 6. Conclusion

The findings of the study offer valuable insights into the ethical stances held by key actors involved in brain data research. This research highlights the myriad challenges and deliberations inherent in the pursuit of a globally informed framework for governing brain data. Moreover, it illuminates several critical considerations that demand thorough examination in the advancement of global brain data governance.

The study provides essential insights, considerations, and recommendations that align with the various dimensions of brain data governance, encompassing human rights, participatory governance, regulations, policies, guidelines, and the promotion of ethical innovation. Employing ethical theories, this research exemplifies how these theories facilitate the balancing of interests among stakeholders across different regions while emphasising the importance of value alignment.

Furthermore, it furnishes normative guidance by synthesising diverse positions, principles, and values that should serve as the foundation for data governance decisions and actions. The insights derived from this research also underscore the role of ethical theories in informing the assessment of potential risks and benefits associated with the processing of brain data, as perceived by neuroscientists.

This research has demonstrated that enhancing an understanding of the ethical stances held by pivotal stakeholders can enhance the nuanced approach to discussions in brain data governance. There's a need to establish day-to-day practices and routines that bring clarity, thus aiding essential stakeholders in effectively navigating the ethical and legal complexities inherent in brain data research. Some crucial considerations encompass fundamental concepts such as data protection by design and by default, and privacy by design (Eke D. et al., 2021). Moreover, ethics by design (European Commission, 2021) and by default stands as another pivotal concept capable of infusing ethics into everyday decision-making processes, thereby fostering ethical adherence. This is particularly valuable as certain perspectives from this study have emphasised the necessity of seamlessly integrating contextual perceptions and concerns into practical implementation of data governance frameworks. The findings show the need to move from recommendations and discussions to practical implementation of solutions to address concerns and to provide ethical and legal clarity which will advance the navigation of current ethical and legal hurdles and advance discussions in the development of a global governance framework.

## Data availability statement

The original contributions presented in the study are included in the article/supplementary material, further inquiries can be directed to the corresponding author.

## Ethics statement

The studies involving humans were approved by De Montfort University CEM Research Ethics Committee. The studies were conducted in accordance with the local legislation and institutional requirements. The participants provided their written informed consent to participate in this study.

## Author contributions

DE: conceptualization, methodology, supervision, and writing—review & editing. PO: conceptualization, data collection and curation, formal analysis, investigation, methodology, validation, writing—original draft, and writing—review & editing. BS: conceptualization, funding acquisition, resources, supervision, and writing—review & editing. All authors contributed to the article and approved the submitted version.
