# Corrigendum: Towards an understanding of global brain data governance: ethical positions that underpin global brain data governance discourse

**DOI:** 10.3389/fdata.2023.1344345

**Published:** 2023-12-19

**Authors:** Paschal Ochang, Damian Eke, Bernd Carsten Stahl

**Affiliations:** ^1^Centre for Computing and Social Responsibility, De Montfort University, Leicester, United Kingdom; ^2^School of Computer Science, University of Nottingham, Nottingham, United Kingdom

**Keywords:** brain data, data governance, neuroethics, ethics, neurodata, ethical theories, ethical positions

In the published article, there was an error in the author list arrangement, the corrected author list appears below.

Paschal Ochang^1*^, Damian Eke^1^ and Bernd Carsten Stahl^1,2^

The authors apologize for this error and state that this does not change the scientific conclusions of the article in any way. The original article has been updated.

